# Expanding the Spectrum of Movement Disorders Associated With *C9orf72* Hexanucleotide Expansions

**DOI:** 10.1212/NXG.0000000000000575

**Published:** 2021-03-12

**Authors:** Carlos Estevez-Fraga, Francesca Magrinelli, Davina Hensman Moss, Eoin Mulroy, Giulia Di Lazzaro, Anna Latorre, Melissa Mackenzie, Henry Houlden, Sarah J. Tabrizi, Kailash P. Bhatia

**Affiliations:** From the Department of Neurodegenerative Diseases (C.E.-F., D.H.M., S.J.T.), Department of Clinical and Movement Neurosciences (A.L, F.M., E.M., G.D.L., M.M., K.P.B.), and Department of Neuromuscular Disorders (H.H.), UCL Queen Square Institute of Neurology, United Kingdom; Department of Neurosciences, Biomedicine and Movement Sciences (F.M.), University of Verona, Italy; St George's University of London (D.H.M.), United Kingdom; Department of Systems Medicine (G.D.L.), University of Rome Tor Vergata, Italy; and Pacific Parkinson's Research Centre and Djavad Mowafaghian Centre for Brain Health (M.M.), University of British Columbia, Vancouver, Canada.

## Abstract

**Objective:**

Hexanucleotide repeat expansions (HREs) in *C9orf72* are a major cause of frontotemporal dementia (FTD) and amyotrophic lateral sclerosis (ALS). We aimed to determine the frequency and phenomenology of movement disorders (MD) in carriers of HRE in *C9orf72* through a retrospective review of patients' medical records.

**Methods:**

We retrospectively reviewed the clinical records of patients carrying a *C9orf72* HRE in the pathogenic range and compared the characteristics of patients with and without MD.

**Results:**

Seventeen of 40 patients with a *C9orf72* HRE had a documented MD. In 6 of 17, MD were the presenting symptom, and in 2 of 17, MD were the sole manifestation of the disease. FTD was present in 13 of 17 patients, ALS in 5 of 17 patients, and 2 of 17 patients did not develop FTD or ALS. Thirteen of 17 patients had more than one MD. The most common MD were parkinsonism and tremor (resembling essential tremor syndrome), each one present in 11 of 17 patients. Distal, stimulus-sensitive upper limbs myoclonus was present in 6 of 17 patients and cervical dystonia in 5 of 17 patients. Chorea was present in 5 of 17 patients, 4 of whom showed marked orofacial dyskinesias. The most frequent MD combination was tremor and parkinsonism, observed in 8 of 17 patients, 5 of whom also had myoclonus. *C9orf72* patients without MD had shorter follow-up times and higher proportion of ALS, although these results did not survive the correction for multiple comparisons.

**Conclusions:**

MD are frequent in *C9orf72*. They may precede signs of ALS or FTD, or even be present in isolation. Parkinsonism, tremor, and myoclonus are most commonly observed.

The GGGGCC hexanucleotide repeat expansion (HRE) in the *C9orf72* gene is the most frequent genetic cause of frontotemporal dementia (FTD) and amyotrophic lateral sclerosis (ALS).^1,2^ Movement disorders (MD) are frequently observed in *C9orf72* HRE carriers,^3^ generally alongside the features of FTD or ALS, with parkinsonism being present in up to 40%.^4^

The prevalence of *C9orf72* HRE in other MD has also been investigated. There is no association between *C9orf72* HRE and Parkinson disease^5^ or essential tremor syndrome (ETS).^6^ Conversely, *C9orf72* HRE seems over-represented in corticobasal syndrome (CBS)^7^ and is the most frequent Huntington disease phenocopy.^8^

In this study, we sought to define the MD associated with HRE in the *C9orf72* gene and compared the characteristics of *C9orf72* patients with and without MD.

## Methods

### Standard Protocol Approvals, Registrations, and Patient Consents

All participants were recruited under ethics-approved research protocol (UCLH: 04/N034). Clinical records of all patients with HRE in *C9orf72* tested at the National Hospital for Neurology and Neurosurgery between May 2012 and May 2019 were retrospectively reviewed. Filmed patients provided written informed consent before video recording.

### Genetic Analysis

Repeat-primed fluorescent PCR (RP-PCR) was performed as previously reported in the study by Renton et al. to test for the presence of an expansion in *C9orf72*.^1^ An ABI (Carlsbad, CA) 3730xl automated sequencer was used for fragment length analysis through capillary electrophoresis. Analysis of RP-PCR electropherograms was performed using Peak Scanner v1.0 (ABI). Expansions with a characteristic sawtooth pattern were put forward for Southern blotting.

Southern hybridization^9^ was performed by combining the use of a (GGGGCC)_5_ probe targeting multiple sites within the expansion and genomic DNA digested with 2 frequently cutting restriction endonucleases whose sites closely flanked the repeat region. Expansion size was estimated by interpolation of autoradiographs using a plot of log_10_ base pair number against migration distance.

A minimal size of 30 GGGGCC repeats in the *C9orf72* gene was defined as pathogenic.^10^ The expansions were classified as “small” if patients had between 30 and 90 GGGGCC repeats and “large” if there were >90 GGGGCC repeats.

### Statistical Analysis

Quantitative data were not normally distributed after visual evaluation of the data and using the Shapiro-Wilk test. Statistical testing was subsequently performed using the Mann-Whitney *U* test. Fisher exact test was used to compare proportions. The results were corrected for multiple comparisons using the Benjamini-Hochberg false discovery rate (FDR), with FDR <0.05 deemed a significant result.

Analyses were performed using Stata version 12.0 (StataCorp, College Station, TX).

### Data Availability

Anonymized data not published in this article will be available from the corresponding author on reasonable request.

## Results

### Patients

A total of 501 patients underwent *C9orf72* HRE testing. Of these, 53 patients had more than 30 repeats. Clinical data were available in 40 of 53 patients. Among the 40 patients with HRE and available data, 17 had MD according to their clinical notes (table 1; figure e-1, links.lww.com/NXG/A394).

**Table 1:**
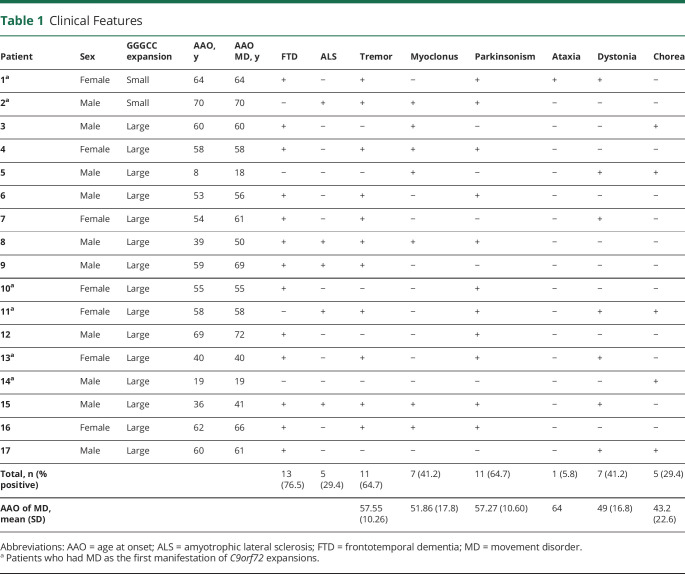
Clinical Features

Patients who developed MD were referred to the neurology clinics because of either MD (7/17) or cognitive symptoms (9/17), or both (1/17). Genetic analysis of *C9orf72* was requested because of the combination of MD and cognitive symptoms (4/17), cognitive symptoms and family history of neurodegenerative conditions (7/17), MD and positive family history (2/17), isolated MD (3/17), and isolated cognitive symptoms (1/17).

Among patients with MD and HRE, *C9orf72* testing was requested from consultant neurologists from the cognitive (8/17), MD (6/17) general neurology (2/17), and neuromuscular clinics (1/17). Of the 17 patients with *C9orf72* HRE and MD, 2 of 17 had small HREs and the remainder had large expansions in *C9orf72*. Seven of 17 patients (41.18%) were women. Median age at onset in patients with MD was 58 years (range 8–70). A family history of dementia or ALS in first-degree relatives was present in 64.7% of the cases with MD and 69.5% of the cases without MD.

### Neuropsychometry

Fourteen of 17 patients with MD had available neuropsychological assessments (table 2). Among them, 10 of 14 presented executive dysfunction and/or memory impairment, whereas 7 of 14 had slow processing speed. Either visuospatial dysfunction, impaired calculation, or attention deficits were present in 2 of 14 patients, whereas 4 of 14 patients had decreased verbal fluency.

**Table 2:**
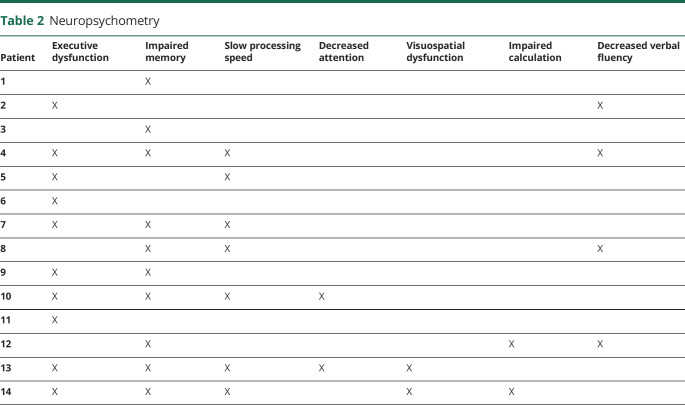
Neuropsychometry

### Neuroimaging

Fifteen of 17 patients had available brain MRI reports. Findings included generalized atrophy in 7 of 15 patients, frontal atrophy in 2 of 15 patients, small vessel disease in 2 of 15 patients, and 1 of 15 patient had midbrain atrophy. The remaining 3 of 15 patients had a normal MRI. No patients exhibited asymmetric atrophy.

### Symptom Onset and Progression

MD were the initial disease manifestation in 6 of 17 patients: 1 presented with cerebellar ataxia, 1 with hemichorea, 1 with cervical dystonia, and 3 with parkinsonism. Among the other 11 patients, 8 presented with cognitive symptoms (7 with FTD and 1 with learning disabilities) and 3 had a psychiatric onset (1 recurrent panic attacks, 1 psychotic episode, and 1 schizoid personality disorder). In patients who did not present with MD, median time from first symptom to development of MD was 3.5 years (range 0–11 years).

During the follow-up, 10 of 17 patients developed FTD, 2 of 17 developed ALS, and 3 of 17 developed FTD/ALS overlap. The 2 patients who continued to exhibit solely MD features at the last follow-up had the earliest age at onset (AAO).

### Movement Disorders

Most patients (13/17) had more than one MD. The most frequent combination was tremor and parkinsonism (8/17 patients), 5 of whom also had myoclonus.

**Figure 1:**
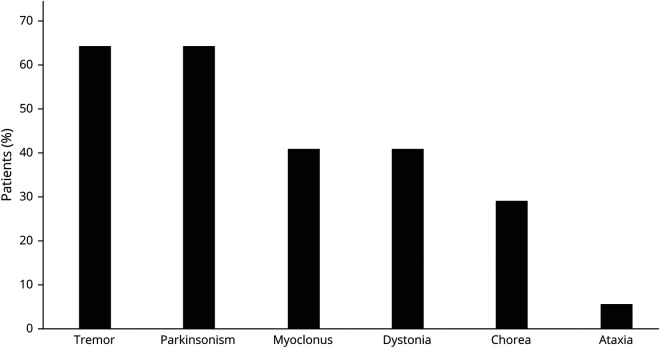
Distribution of Movement Disorders in Patients With Hexanucleotide Repeat Expansions in *C9orf72*

Tremor and parkinsonism were equally prevalent MD manifestations in this cohort, each one affecting 11 of 17 patients (table 1; figure 1). Phenomenologically, tremor usually resembled ETS (8/11) as low-amplitude, high-frequency postural and intention tremor affecting the upper limbs symmetrically. Other phenotypes, occasionally overlapping with ETS-like tremor, included “jerky” arm tremor (2/11), rest tremor affecting the arms alone (2/11) or legs with later progression to the arms (3/11), tongue tremor (1/11), and isolated “no-no” head tremor (1/11).

Parkinsonism was mild in 9 of 11 patients and severe and rapidly progressive in the remaining 2 patients, leading them to become bedbound within a year from onset. Parkinsonism was asymmetric in 6 of 11 patients; 3 of 11 patients received a clinical diagnosis of CBS (video 1, segment 1, links.lww.com/NXG/A396). DaTscan was performed in 3 of 11 patients and was abnormal in all. Levodopa was administered to all 3 patients with abnormal DaTscan, providing subjective benefit in 2. One patient without available DaTscan did not improve with levodopa.

10.1212/000575_Video_1 Video 1 Segment 1: a patient presenting with facial hypomimia, bradykinesia, ideomotor apraxia, and stimulus-sensitive distal myoclonus in the upper limbs. Segment 2: a patient showing orofacial dyskinesias, ideomotor apraxia, and chorea in his upper limbs.Download Supplementary Video 1 via http://dx.doi.org/10.1212/000575_Video_1

Myoclonus was present in 7 of 17 patients. In most (6/7 patients), this manifested as distal stimulus-sensitive jerks affecting both upper limbs (video 1, segment 1, links.lww.com/NXG/A396), whereas one patient was considered to have isolated myoclonus of the cheek by a MD specialist.

Dystonia was observed in 7 of 17 patients during the follow-up. In 5 of 7, it exclusively involved the cervical region, whereas one patient had hemidystonia and another had dystonic posturing of the arms.

Five patients had chorea, which was limited to the perioral region in 2 patients, to one hemibody in another, and generalized in 2. There were marked orofacial dyskinesias in 4 of 5 patients with chorea (video 1, segment 2, links.lww.com/NXG/A396) resembling tardive dyskinesia, although only one patient had a previous history of neuroleptic exposure. One patient had prominent appendicular ataxia.

Seizures occurred in 4 of 17 patients. Further details are available in the table e-1 (links.lww.com/NXG/A395).

### Comparison Between *C9orf72* Patients With and Without MD

Patients with HRE and MD (MD+) had a lower proportion of ALS compared with patients with HRE without MD (MD−) (5/17 in MD+ vs 17/23 MD−), a higher proportion of FTD (13/17 MD+ as opposed to 11/23 MD−) and longer median follow-up times (8 years in MD+ compared with 3 years in MD−). Median time from onset to death was longer in the patients with HRE and MD than in those without MD (8 years in MD+ vs 2.5 years in MD−). A family history of neurodegenerative disorders did not differ between cohorts (11/17 MD+ vs 16/23 MD−) (table 3). None of this results survived correction for multiple comparisons at a *p*_FDR_ = 0.05.

**Table 3:**
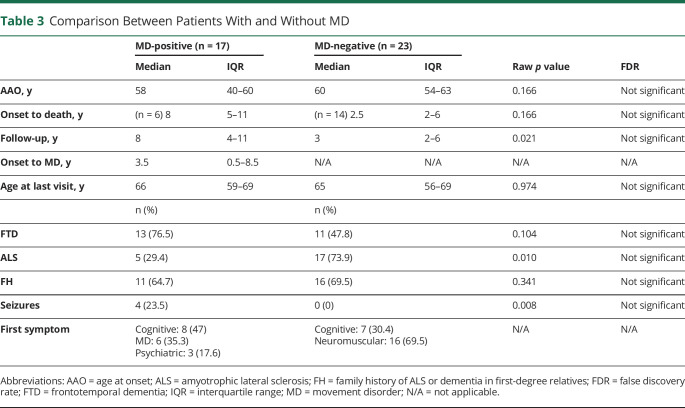
Comparison Between Patients With and Without MD

## Discussion

This study examined the MD associated with HRE in *C9orf72* and the differences between HRE carriers with and without MD. Our findings suggest that MD are frequent in patients with HRE in *C9orf72* and often precede FTD/ALS. Moreover, MD can be the initial or even the sole manifestation of *C9orf72* HRE. Consequently, the spectrum of MD may be significantly more diverse than previously appreciated.

Tremor and parkinsonism were the most frequent MD in our cohort. Although rest tremor has previously been reported in HRE carriers with parkinsonism, postural and intentional tremor, frequently observed in our study, were only mentioned in one previous study as being rare.^4,8^

Parkinsonism is a well-recognized feature of the *C9orf72* HRE syndrome, particularly in those with FTD phenotypes. However, in 2 patients, levodopa-unresponsive parkinsonism was the cardinal clinical feature and exhibited a rapidly progressive course, which has not previously been reported.

Symmetric, stimulus-sensitive upper limb myoclonus, frequently coexisting with parkinsonism, and chorea with marked orofacial involvement were also a common finding in our cohort.

Despite MD being frequent in *C9orf72* HRE carriers, the phenotypic manifestations do not fall neatly into other syndromic categories, e.g., progressive supranuclear palsy and multiple system atrophy. It is therefore unlikely that misdiagnosis as neurodegenerative entities other than FTD or ALS would occur.^2^ This perhaps explains the low frequency of *C9orf72* HRE among patients with clinical diagnoses of other neurodegenerative conditions.^7,11^

None of the contrasts between *C9orf72* HRE with and without MD survived multiple comparisons correction. This may be related to the small sample sizes in both cohorts (MD+ and MD−), which is inherent to the low frequency of *C9orf72* expansions. However, MD were less common in those presenting with ALS phenotypes. This may simply reflect shorter follow-up times and difficulty in identifying certain MD in the context of ALS.^1,2,10^ Of note, the 2 sole patients who did not develop FTD or ALS had the earlier AAO, being therefore possible that either of these conditions is developed during the follow-up.

Our study has several limitations, mainly derived from its retrospective nature. Video recordings were only available in 2 patients, information about previous medications was not systematically recorded in medical notes, and none of our patients had neurophysiologic studies performed. Furthermore, most of the patients were unable to visit our hospital for a clinical review. Clinical data therefore had to be obtained from the medical record and/or discussion with the treating consultant whenever necessary.

Our results suggest that *C9orf72*-associated MD are clinically heterogeneous and frequently found in combination. ETS-like tremor, parkinsonism, distal myoclonus, chorea with orofacial involvement, and cervical dystonia in the context of familial neurodegenerative conditions should prompt molecular genetic testing of the *C9orf72* gene. Further prospective, systematic studies are needed to accurately assess the phenotypic manifestations of *C9orf72*-associated MD.

## Glossary

AAO: age at onset
ALS: amyotrophic lateral sclerosis
CBS: corticobasal syndrome
ETS: essential tremor syndrome
FDR: false discovery rate
FTD: frontotemporal dementia
HRE: hexanucleotide repeat expansion
MD: movement disorder
